# Research Progress of Pharmacogenomics in Drug-Induced Liver Injury

**DOI:** 10.3389/fphar.2021.735260

**Published:** 2021-09-06

**Authors:** Qihui Shao, Xinyu Mao, Zhixuan Zhou, Cong Huai, Zhiling Li

**Affiliations:** ^1^ Department of Pharmacy, Shanghai Children’s Hospital, Shanghai Jiao Tong University, Shanghai, China; ^2^ Shanghai Jiao Tong University School of Medicine, Shanghai, China; ^3^ Bio-X Institutes, Key Laboratory for the Genetics of Developmental and Neuropsychiatric Disorders (Ministry of Education), Shanghai Jiao Tong University, Shanghai, China

**Keywords:** pharmacogenomics, drug-induced liver injury, gene polymorphism, precision medicine, pharmacogenetics

## Abstract

**Background:** Drug-induced liver injury (DILI) is a common and serious adverse drug reaction with insufficient clinical diagnostic strategies and treatment methods. The only clinically well-received method is the Roussel UCLAF Causality Assessment Method scale, which can be applied to both individuals and prospective or retrospective studies. However, in severe cases, patients with DILI still would develop acute liver failure or even death. Pharmacogenomics, a powerful tool to achieve precision medicine, has been used to study the polymorphism of DILI related genes.

**Summary:** We summarized the pathogenesis of DILI and findings on associated genes and variations with DILI, including but not limited to HLA genes, drug metabolizing enzymes, and transporters genes, and pointed out further fields for DILI related pharmacogenomics study to provide references for DILI clinical diagnosis and treatment.

**Key Messages:** At present, most of the studies are mainly limited to CGS and GWAS, and there is still a long way to achieve clinical transformation. DNA methylation could be a new consideration, and ethnic differences and special populations also deserve attention.

## Introduction

Drug induced liver injury (DILI) refers to the liver injury induced by all kinds of prescription or non-prescription chemical drugs, biological agents, traditional Chinese medicine, natural medicine, health care products, dietary supplements and their metabolites or even excipients (Yu et al., 2017). Once DILI occurs clinically, medication must be stopped or modified. Drug induced liver injuries are classified into dose-dependent type and dose-independent type, or also named intrinsic type and idiosyncratic type, respectively. The majority of DILIs in clinical settings are idiosyncratic DILI and the mechanism of DILI is still under study. The onset of intrinsic DILI is caused by the direct toxicity of drugs or their metabolites, so it is predictable and the incubation period is short (several hours to several days); the onset of idiosyncratic DILI is often unpredictable and the incubation period is uncertain (Katarey and Verma, 2016; European Association for the Study of the Liver, 2019), which cannot be predicted or simulated by animal models. Patients with DILI generally suffer from loss of appetite, fatigue, upper abdominal discomfort, nausea and other digestive symptoms. When the disease aggravates, patients may develop ascites, coagulation disorders, cirrhosis, hepatic encephalopathy, etc. In addition, if DILI patients are not treated in time and progress to chronic DILI, the damaged liver cells can no longer be completely recovered even with systematic treatments, which may result in cholestasis, hepatitis, cirrhosis, etc. Shen et al. (2019) conducted a retrospective study on patients with DILI in multiple regions in China from 2012 to 2014 and found that 51.39% of DILI patients had liver cell injury and 28.30% had mixed liver injury, among which 13% had chronic DILI and the mortality rate was 0.39%. It is estimated that the annual incidence of DILI in China is about 23.80/100,000, higher than that in western countries. A population-based study in Iceland in 2013 demonstrated an incidence of 19 cases per 100,000 per year (Björnsson et al., 2013), and a study based on hospitalized cases in university hospitals conducted in South Korea estimated that the incidence rate in South Korea was 12/100,000 persons/year (Suk et al., 2012), which are all lower than that in China. Due to the harmfulness and prevalence of DILI, it is urgent to develop new technologies to prevent DILI.

As DILI often has an insidious onset and generally lacks specific clinical manifestations, biomarkers, imaging, and pathomorphological features, the Roussel UCLAF Causality Assessment Method (RUCAM) scale has been established for DILI diagnosis. RUCAM is currently the most widely used and recognized tool for the causality assessment methods for DILI. It can be applied to both individuals and prospective or retrospective studies and more than 81,000 cases of special DILI have been assessed with the RUCAM scale so far (Teschke and Danan, 2020). Clinically, when patients exhibit abnormal elevation of serum ALT and or ALP [ALT greater than 5 times the upper limit of normal (ULN) and (or) ALP≥2×ULN, with/without other clear evidence of liver damage] after drug treatment, doctors will stop the use of drug and apply RUCAM for causality assessment. Considering the patient’s age, gender, and medical history, the RUCAM scale estimates the relationship between liver injury and the usage of drug, and classifies the subtype of DILI. The R value [R=(ALT/ULN)/(ALP/ULN)] is used as a classification indicator. Misdiagnosis rate can be reduced by RUCAM but not completely eliminated. In most cases, clinical patients still choose to pay attention to the changing trend of hepatic enzymatic indexes to judge the degree of liver damage caused by drugs. Therefore, all cases mentioned in this review are based on exceeding of ALT/ALP to determine liver damage on the basis of excluding basic diseases and virus infection.

Pharmacogenomics (PGx) is a subject to study the different reactions to drugs of the population, which are caused by genetic variation and polymorphisms, so as to predict the risk of adverse reactions to drugs (Wake et al., 2019). Genetic variations affect the expressions or activities of metabolic enzymes, drug receptors and transporters, thereby increasing the risk of adverse reactions or reducing therapeutic effects, so PGX can be used as a guidance of drug treatment in clinical. Understanding the pathogenesis of DILI is conducive to understanding the important role of related genes in DILI, and understanding the correlation between DILI and the polymorphism of related genes can assist in clinical assessment of the risk of DILI and auxiliary diagnosis of idiosyncratic DILI.

## Pathogenesis of DILI

**FIGURE 1:**
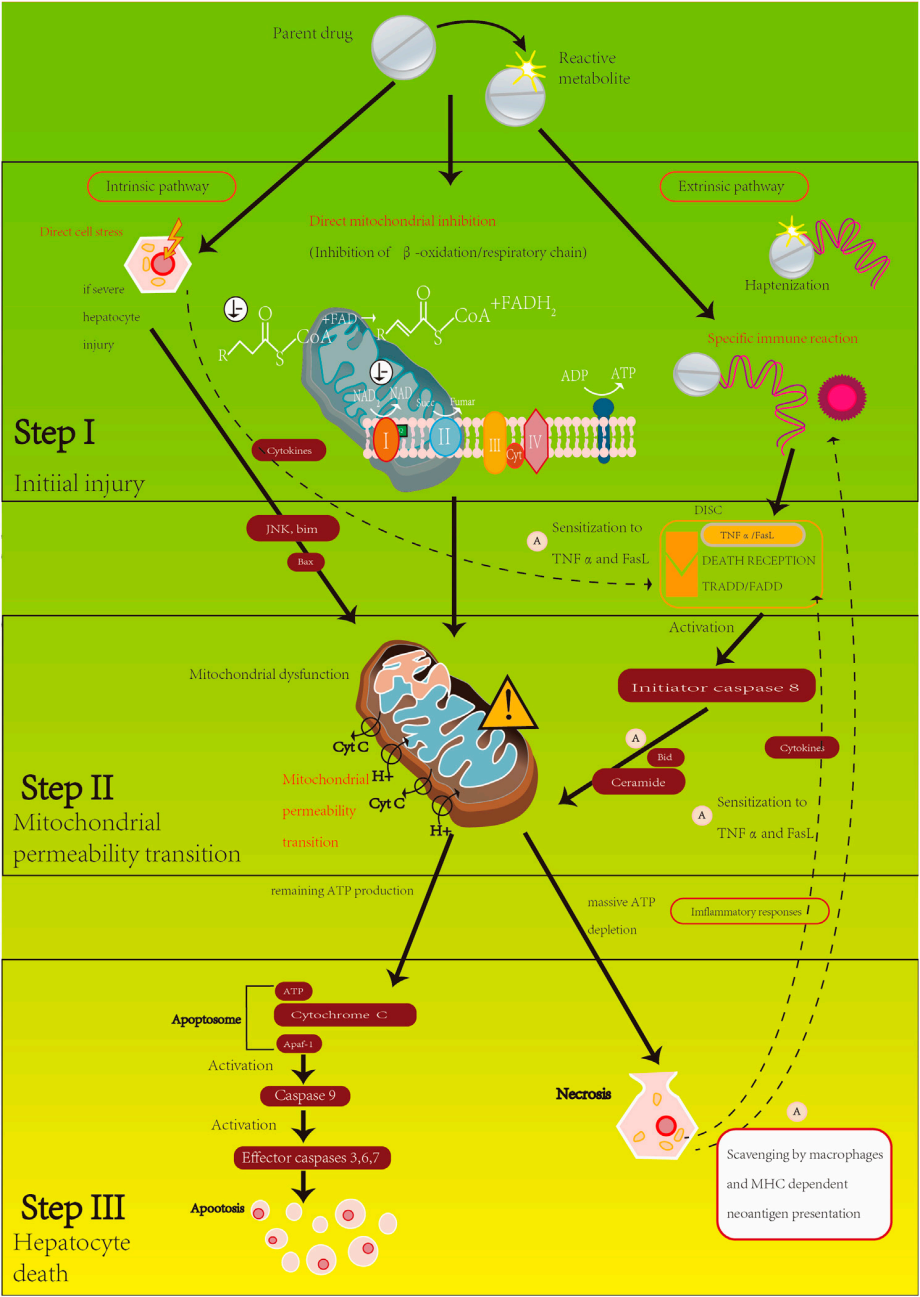
Pathogenesis of DILI.

The pathogenesis of DILI is very complicated, since it is often the result of multiple pathways. There are several hypotheses on the pathogenesis of DILI; among them, the “three-step mechanism” (Russmann et al., 2010) is the most widely recognized (Figure 1). This hypothesis suggests that DILI is a progressive process that includes initial damage, mitochondrial damage, and cell death. Initial damage can lead to further mitochondrial permeability transformation (MPT) and eventually to apoptosis or necrosis of hepatocytes (Galluzzi et al., 2016). It can be caused by various events, such as direct cell stress, direct mitochondrial damage, specific immune response, etc. Drugs and their metabolites can cause direct cellular stress and specifically impair mitochondrial function. Excessive medication or genetic abnormality of drug metabolism enzymes will produce a large amount of active metabolites such as free radicals, which will deplete glutathione in the liver and combine with the unsaturated fatty acids of cell membrane phospholipids, resulting in peroxidation that can lead to membrane damage, ATP depletion, mitochondria damage, and liver cell necrosis. Besides, mitochondrial dysfunction in liver cells is also a major cause of initial damage. DNA damage and consumption, increased oxidative stress, and permeability changes are all manifestations of drug interference with mitochondrial function (Hu and Lou, 2020). Mitochondrial dysfunction is also directly related to the activation of some oxidative stress signaling pathways, including the mitogen-activated protein kinase (MAPK) signaling pathway (Ren et al., 2017) and the c-Jun amino-terminal kinase (JNK) pathway (Seki et al., 2012). In addition, some drugs can activate the liver’s innate immune system, leading to autoimmune hepatitis (AIH), in which the “hapten hypothesis” is crucial. The drug metabolites in hepatocytes and peptides form drug-peptide complexes, which are recognized by human leukocyte antigen (HLA) class II molecules through antigen presentation and then interact with cluster of differentiation 4 (CD4) positive T cells. The T cell receptor (TCR) on the CD4^+^ T cells interacts to activate the downstream surface antigen differentiation cluster 8 (CD8) positive T cells and B cells, thereby producing a large number of specific antibodies and autoantibodies, which in turn lead to liver damage (Zhan et al., 2019). Some HLA allele mutations can increase the susceptibility of liver injury to specific drugs, which supports this hypothesis.

MPT can be caused not only by initial damage (such as cell stress and specific immune response), but also by the combination of drugs and their metabolites with death receptors (such as tumor necrosis factor (TNF) superfamily) that leads to the external pathway of apoptosis activation (Teng et al., 2011). In external pathways, mild initial damage may be amplified. Mild stress or other factors can make hepatocytes more susceptible to death receptors such as TNF by innate immune system regulation.

Damage to mitochondrial function will eventually lead to cell apoptosis or necrosis. A large influx of protons will be caused by MPT through inner mitochondrial membrane and prevent the synthesis of ATP. Once mitochondrial ATP is depleted, the basement membrane expands and outer membrane permeability increases, which will enable the pro-apoptotic substances such as cytochrome C to enter the cell and cause apoptosis.

## DILI and Gene Polymorphism

The onset and extent of DILI varies drastically in different patients using identical drugs and the individual differences are largely due to genetics. At present, candidate gene studies (CGS) and genome-wide association studies (GWAS) have shown that specific DILI is associated with genetic susceptibility of genes.

### HLA Gene

Major histocompatibility complex (MHC) is a group of genes that determine the compatibility of transplantation and are closely linked with immune response. Human MHC, also known as HLA gene complex, encodes HLA molecules or HLA antigens. It is located on chromosome 6, which consists of more than 200 genes, and can be divided into three subgroups: Class I, Class II, and Class III. Class I MHC molecules are recognized by CD8^+^ T cells and consist of *HLA-A*, *HLA-B* and *HLA-C* genes. Class II MHC molecules are recognized by CD4^+^ T cells and consist of *HLA-DPA1*, *HLA-DPB1*, *HLA-DQB1*, *HLA-DRA,* and *HLA-DRB1*. Class III MHC mainly encodes complement components, tumor necrosis factor (TNF), heat shock protein 70 (HSP70) and 21 hydroxylase gene (*CYP21A* and *CYP21B*) (Fan et al., 2017). Polymorphisms of HLA genes identified through GWAS and CGS studies as involved in DILI susceptibility are outlined in Table 1.

**TABLE 1:** Polymorphisms of DILI-related HLA genes identified in GWAS and CGS.

Genes	Drug	Method	Cohort	OR	P	95%Cl
A*02:01 rs2523822 TRNAI25	Amoxicillin-clavulanate	GWAS	201 cases; 531 controls (European) Lucena et al. (2011)	2.3	1.8 × 10^–10^	1.8–2.9
A*30:02	Amoxicillin-clavulanate	CGS	75 cases; 885 controls (Spanish) Stephens et al. (2013)	6.7	2.6 × 10^−6^	2.8–15.9
A*33:01	Terbinafine	GWAS	21 cases; 10,588 controls (European) Nicoletti et al. (2017)	40.5	6.7*10^−10^	12.5−131.4
A*33:01	Fenofibrate	GWAS	7 cases; 10,588 controls (European) Nicoletti et al. (2017)	58.7	3.2 × 10^−7^	12.3−279.8
A*33:01	Ticlopidine	GWAS	5 cases; 10,588 controls (European) Nicoletti et al. (2017)	163.1	0.00002	16.2−1642
A*33:03	Ticlopidine	CGS	22 cases; 85 controls (Japanese) Hirata et al. (2008)	13.0	1.24 × 10^−5^	4.40–38.59
B*14:01	Compound sulfamethoxazole	GWAS	51 cases; 12,156 controls (European and American) Li et al. (2021)	9.2	0.0003	3.16–22.35
B*18:01	Amoxicillin-clavulanate	CGS	75 cases; 885 controls (Spanish) Stephens et al. (2013)	2.9	0.0082	1.3 ± 6.2
B*35:01	Polygonum multiflorum	CGS	26 cases; 99 controls (Chinese) Li et al. (2019)	143.9	4.8 × 10^−10^	30.1–687.5
B*35:02	Minocycline	GWAS	25 cases; 10,588 controls (European) Urban et al. (2017)	29.6	2.5 × 10^−8^	7.8–89.8
B*39:01	Infliximab	GWAS	18 cases; 60 controls (white person) Bruno et al. (2020)	43.6	0.001	2.8-+∞
B*57:01	Flucloxacillin	GWAS	197 cases; 6835 controls (European) Nicoletti et al. (2019)	36.6	2.67 × 10^−97^	26.14–51.29
B*57:01	Pazopanib	GWAS	429 cases; 1761 controls (Global) Xu et al. (2016)	2.0	0.0014	1.3–3.1
B*57:03	Flucloxacillin	GWAS	197 cases; 6835 controls (White person) Nicoletti et al. (2017)	79.2	1.2 × 10^−6^	3.37–116.1
B*58:01	Nevirapine	CGS	57 cases; 111 controls (African) Hirata et al. (2008)	U	U	U
DRB1*01:02	Nevirapine	CGS	57 cases; 111 controls (African) Phillips et al. (2013)	U	U	U
DRB1*07	Ximelagatran	GWAS	74 cases; 130 controls (European) Kindmark et al. (2008)	4.4	U	U
DRB1*07:01	Lapatinib	GWAS	37 cases; 1071 controls (European) Schaid et al. (2014)	14	2.4 × 10^−13^	6.36–31.32
DRB1*16:01-DQB1*05:02	Flupirtine	GWAS	614 cases; 10,588 controls (European) Nicoletti et al. (2016)	18.7	0.002	2.5–140.5
DRB1*15:01- DQB1*06:02	Amoxicillin-clavulanate	CGS	75 cases; 885 controls (Spanish) Stephens et al. (2013)	3.0	5.1 × 10^−4^	1.6 ± 5.5

OR, odds ratio; P, *p* value; 95%CI, 95% confidence interval; C, medication with adverse reactions; T, medication with no adverse reactions; PC, population control; U, unknown.

Studies have shown that antibiotics account for the largest proportion of idiosyncratic DILI (Katarey and Verma, 2016). Among antimicrobial agents, amoxicillin-clavulanate were the first drugs found to induce DILI by certain HLA polymorphism, and many HLA genotypes have been found to increase the hepatotoxicity of amoxicillin. HLA-A*02:01, HLA-A*30:02 and other gene carriers increased susceptibility to amoxicillin-DILI, therefore these genes can be used as biomarkers for auxiliary diagnosis of DILI (Lucena et al., 2011; Stephens et al., 2013). In addition to amoxicillin, the correlation between HLA genes and hepatotoxicity induced by other antimicrobial agents has been widely studied. HLA-B*57:01 is the main risk factor for flucloxacillin-DILI and HLA-B*57:03 also show correlation (Nicoletti et al., 2019). HLA-B*14:01 was associate with liver injury induced by cotrimoxazole (Li et al., 2021), while HLA-B*35:02 increase the risk of the occurrence of DILI caused by minocycline (Urban et al., 2017). Among antifungal drug, HLA-A*33:01 was significantly correlated with terbinafine induced DILI (Nicoletti et al., 2017). For antiviral drugs, both HLA-B*58:01 and HLA-DRB1*01:02 increase the susceptibility to liver injury induced by nevirapine (Phillips et al., 2013).

Besides, central nervous system inhibitors, antineoplastic drugs, and analgesics can also lead to idiosyncratic DILI (Katarey and Verma, 2016). In cardiovascular preparations, in addition to HLA-A*33:01 that is associated with fenofibrate and ticlopidine, HLA-A*33:03 may increase the risk of ticlopidine-induced DILI (Hirata et al., 2008). HLA-DRB1*07 has been confirmed to be associated with Ximelagatran-induced DILI (Kindmark et al., 2008). Among antitumor drugs, recent studies (Bruno et al., 2020) found that multiple HLA alleles were associated with DILI induced by infliximab, and HLA-B*39:01 was identified as a potential risk factor for DILI induced by infliximab; other studies (Spraggs et al., 2011; Schaid et al., 2014; Xu et al., 2016) showed that HLA-B*57:01 and HLA-DRB1*07:01 were associated with DILI induced by pazopanib and lapatinib, respectively. Among the analgesics, DRB1*16:01-DQB1*05:02 was found to be associated with Flupirtine-related liver injury, with a 19-fold increased risk of clinically significant adverse events in people with this haplotype (Nicoletti et al., 2016).

Drug-induced liver injury caused by traditional Chinese medicine and dietary supplements has attracted more and more attention in recent years (Katarey and Verma, 2016). Polygonum multiflorum (PM) is a kind of traditional Chinese medicine widely used in the world, which has been used to tonify the kidney and nourish the liver since nearly a thousand years ago in China. At present, PM is still found in many herbal prescriptions, dietary supplements and natural drug products in some countries in Asia, Europe and America. With the increasing incidence of PM-induced DILI, the research on PM-DILI has been further deepened. The known HLA-B*35:01 allele is one of the risk factors of PM-DILI and a potential biomarker to assist in the diagnosis of PM-DILI, which was confirmed through a prospective cohort study of 72 patients treated with PM for 4 weeks and an independent replication study of 15 PM-DILI patients, 33 other DILI patients, and 99 population controls (Li et al., 2019).

### Drug Metabolizing Enzymes

Drugs taken by the human body are mainly metabolized by the liver, which involves a variety of reactions and multiple drug metabolizing enzymes. Phase I metabolic reaction is the rate-limiting step for the elimination of drugs from the body, which can cause detoxification or poisoning effects. Then through phase II metabolic reaction, the drug and its metabolites are combined with endogenous substances and discharged from the body. Although this process has a detoxification effect, some active metabolites produced at the same time may also cause liver damage. The biological functions of drug-metabolizing enzymes and the inter-individual variations of related genes make them possible targets of DILI susceptibility. Polymorphisms of drug metabolizing enzymes identified through GWAS and CGS studies as involved in DILI susceptibility are outlined in Table 2.

**TABLE 2:** Polymorphisms of DILI-related drug metabolizing enzymes genes identified in GWAS and CGS.

Genes	Drug	Method	Cohort	OR	P	95%Cl
CYP1A1	Nevirapine	CGS	U (European) Singh et al. (2017)	3.68–4.91	0.13–0.21	U
CYP2A6*4	Valproic acid	CGS	807 cases (Global) Yoon et al. (2020)	0.48	0.01	0.10–0.86
CYP2A6*1/*4	Valproic acid	CGS	79 cases, 200 T (Chinese) Zhao et al. (2017)	2.5	0.035	1.06–5.69
CYP2A6*4/*4				20.27	0.006	2.38–172.62
CYP2B6 rs7254579	Ticlopidine	CGS	22 cases92 T (Japanese) Ariyoshi et al. (2010)	2.1	U	U
CYP2B6*6	Efavirenz	CGS	41 cases, 160 P (African) Yimer et al. (2012)	3.3	U	U
CYP2C9 rs1057910 and rs1799853	Nevirapine	CGS	U (European) Singh et al. (2017)	1.78	0.35	U
	Valproic acid	CGS	807 cases (Global) Yoon et al. (2020)	0.70	0.002	0.25–1.15
CYP2D6	Anti-TB drugs	GWAS	114 cases, 114 controls (Chinese) Wei et al. (2020)	9.193	<0.001	3.624–25.888
CYP2E1				4.390	<0.001	1.982–9.724
CYP2E1 rs203192	Anti-TB drugs	CGS	49 cases, 21 cases (Chinese) Huang et al. (2003)	2.52	U	U
		GWAS	2225 cases, 4906 controls (Global) Cai et al. (2012)	1.36	0.12	0.92–2.00
UGT2B7 rs7439366	Anti-TB drugs	CGS	182 cases, 13 controls (Chinese) Shi et al. (2014)	2.01	U	U
UGT2B7 rs7439366	Diclofenac	CGS	24 cases, 48 T, 122 P (European) Daly et al. (2007)	C *vs.* T: 7.7	0.026	1.1–69.9
				C *vs.* P: 8.5		
GSTM1/GSTT1(null)	Tacrine	CGS	18 cases, 123 controls (European) Simon et al. (2000)	2.3	U	U
	Troglitazone	CGS	25 cases, 85 controls (Japanese) Watanabe et al. (2003)	3.692	0.008	1.354–10.066
	Antimicrobial agents	CGS	44 cases, control data U (Spanish) Okada et al. (2011)	3.52	0.002	1.56–8.22
	Anti-infective Drugs	CGS	49 cases, control data U (Spanish) Lucena et al. (2008)	3.12	0.006	1.37–7.11
	NSAIDs	CGS	19 cases, control data U (Spanish) Lucena et al. (2008)	5.61	0.001	1.99–16.0
	Anti-TB drugs	CGS	50 cases, 246 controls (Indian) Gupta et al. (2013)	7.18	0.007	1.7–32.6
NAT2 slow acetylation type	Anti-TB drugs	GWAS	100 cases, 210 controls (Indonesian) Yuliwulandari et al. (2019)	3.64	0.000	2.21–6.00
		CGS	1527 cases, 7184 controls (Global) Zhang et al. (2018b)	3.15	0.000	2.58–3.84
		CGS	1527 cases, 7184 controls (Western Asian) Davidson et al. (2008)	6.42	U	2.41–17.10
		CGS	1527 cases, 7184 controls (European) Zhang et al. (2018b)	2.32	U	0.58–9.24

OR, odds ratio; P, *p* value; 95%CI, 95% confidence interval; C, medication with adverse reactions; T, medication with no adverse reactions; PC, population control; U, unknown; Anti-TB drugs, Antituberculosis drugs; NSAIDs, Non-steroidal anti-inflammatory drugs.

#### Phase I Drug Metabolizing Enzyme

Phase I metabolic enzymes are mainly involved in the oxidation, reduction and hydrolysis reactions of drugs. The cytochrome P450 superfamily (CYP) is considered to be the most important enzyme, so the role of CYP gene polymorphism on DILI deserves attention.

In the CYP1 family, patients with *CYP1A1m1 rs4646903* genotype and combined genotype TC+CC have a significantly higher risk and severity of DILI after receiving nevirapine treatment (Singh et al., 2017). In addition, *CYP1A2 rs2069514* is closely related to the theophylline clearance rate (Zhang et al., 2018a), which can slow down its metabolism and accumulation in the body, so the frequency of DILI is higher than other patients.

The CYP2 family is the most important research area of the correlation between gene polymorphism and DILI. It can be divided into five subfamilies. Regarding CYP2A subfamily, Chinese people with the deletion of the CYP2A6 gene are more likely to have elevated transaminase after valproate treatment (Zhao et al., 2017; Yoon et al., 2020), which significantly increases the susceptibility of liver injury in patients with epilepsy caused by valproic acid (Tanner and Tyndale, 2017). Regarding CYP2B subfamily, the fast metabolic *CYP2B6 rs7254579* can increase the susceptibility to ticlopidine-induced liver injury (Ariyoshi et al., 2010), while the slow metabolic CYP2B6 (*6/*6) can increase the susceptibility to Efavirenz-induced liver injury by reducing the activity of metabolic enzymes (Yimer et al., 2012). Regarding the CYP2C subfamily, most of the early studies focused on *CYP2C8* gene mutations. But a recent meta-analysis of six studies (involving 807 patients) indicates that individuals carrying *CYP2C9 rs1057910* and *rs1799853* mutations may suffer from liver toxicity induced by valproate (Yoon et al., 2020), especially when the drug is taken with alcohol and nevirapine. Besides, the loss of *CYP2C19 rs4244285* and *rs4986893* gene functions may cause clopidogrel resistance and anti-tuberculosis drug-induced liver injury (Yang et al., 2019). Although the former has not been reported about DILI, the latter is highly correlated with DILI. Regarding the CYP2D subfamily, *CYP2D6* not only increases the risk of liver damage from anti-tuberculosis drugs in Chinese populations (Hu et al., 2018; Wei et al., 2020), but also causes excessive accumulation of tramadol bioactive metabolites, which can increase oxidative stress and cause liver toxicity (Qin et al., 2009; Arafa and Atteia, 2018). Regarding the CYP2E subfamily, the correlation between *CYP2E1* gene and anti-tuberculosis DILI is a hot spot. Compared with genotypes including allelic variants, the *CYP2E1 rs2031920* variant genotype can lead to higher *CYP2E1* activity, resulting in increased levels of hepatotoxic metabolites of anti-tuberculosis drugs (especially isoniazid) (Huang et al., 2003; Tang et al., 2012).

In the CYP3 family, although *in vitro* hepatotoxicity model experiments of drugs related to acute liver failure indicate that high *CYP3A4* activity is a risk factor (Cai et al., 2012), its allele mutation rate is low (Werk and Cascorbi, 2014). Therefore, the correlation between *CYP3A4* gene polymorphism and DILI is relatively low.

#### Phase II Drug Metabolizing Enzyme

UDP-glucuronosyltransferases (UGT) are mainly involved in the metabolism of glucuronic acid and has detoxification effects on both internal and external compounds. Individuals with *UGT1A6* (Hao et al., 2011) and *UGT2B7* (Shi et al., 2014) genetic polymorphisms are at increased risk of liver injury after receiving anti-tuberculosis chemotherapy. The slow metabolites of *UGT1A6* and *UGT1A9* are related to the liver injury induced by tolcapone (Alfirevic and Pirmohamed, 2012), which may be due to the decrease of enzyme activity and the accumulation of toxic substances. In addition, the *UGT2B7 rs7439366* allele is associated with an increased risk of diclofenac-induced liver damage (Daly et al., 2007; Petros et al., 2017), and the mechanism may be the allelic variation leading to the formation of active diclofenac metabolites that trigger DILI.

Glutathione S-transferase (GST) mainly catalyzes the combination of reduced glutathione and exogenous substrates (including intermediate drug metabolites) to promote cell excretion. The research on GST of genetic type DILI mainly focused on *GSTT1* and *GSTM1* (Bhattacharjee et al., 2013). A research indicates that the risk of DILI in carriers with double *GSTT1/M1* null genotype is 2.70 times higher than that of non-carriers (Lucena et al., 2008), after analyzing the genomes of 154 patients diagnosed with DILI. Moreover, the effects of tacrine (Simon et al., 2000), troglitazone (Watanabe et al., 2003; Okada et al., 2011) and other drugs (Gupta et al., 2013) on DILI have been identified.

N-acetyltransferase (NAT) is a metabolic enzyme involved in the acetylation reaction. *NAT2* is mainly involved in all aspects of the metabolism of isoniazid, which is also a hot spot in the study of drug-induced liver injury during anti tuberculosis (anti-TB) in the past 20 years. The polymorphism of *NAT2* determines its acetylation polymorphism, so it can be divided into fast acetylation type, intermediate acetylation type and slow acetylation type. Studies have shown that carriers of *NAT2* slow acetylation genotype are generally more susceptible to hepatotoxicity induced by isoniazid anti-tuberculosis drug regimens. This is similar to CYP2E1, but the effect is relatively small (Du et al., 2013; Yuliwulandari et al., 2019; Zhang et al., 2020). It is also worth noting that there are also variants of slow acetylation of NAT2 (Zhang et al., 2018b). For example, NAT2*6/*7 and NAT2*5/*6 genotypes have the highest and lowest risk of ATLI respectively among all slow *NAT2* acetylators combined (Cai et al., 2012). However, these associations are different in different ethnic groups, and the correlation in Asian populations is significantly higher than that in European populations.

### Transporter

In addition to the drug-metabolizing enzymes involved in the biotransformation process, there are also rich and diverse transporters in the liver. In the case of genetic factors change, pathological states, or the presence of drugs, the expression of transporters will change. This will affect the drug concentration in the body and the degree of response of the body, and hence affect the metabolism and lead to the occurrence and development of the disease. Therefore, the expression of transporters plays an important role in the occurrence and development of DILI. Among them, ATP-binding cassette (ABC) transporters and solute carriers (SLC) are particularly important. Polymorphisms of transporters identified through GWAS and CGS studies as involved in DILI susceptibility are outlined in Table 3.

**TABLE 3:** Polymorphisms of DILI-related transporter genes identified in GWAS and CGS.

Genes	Drug	Method	Cohort	OR	P	95%Cl
ABCB1 rs1045642 (3435C→T)	Nevirapine	CGS	9 cases, 49 P (American) Ritchie et al. (2006)	0.254	0.021	0.09–0.76
ABCB1 rs2032582	Nevirapine		30 cases, 414 P (Japanese) Fukunaga et al. (2016)	2.59	6.8 × 10^−4^	1.49–4.56
ABCC2 rs717620 (24C→T)	Diclofenac		24 cases, 48 T, 112 P (English) Daly et al. (2007)	C *vs.* PC: 6.3	C *vs.* PC: 2*10^−4^	C *vs.* PC: 2.38–16.7
				C *vs.* T: 5.0	C *vs.* T: 0.005	C *vs.* T: 1.71–14.7
SLCO1B1 rs4149056	HD-MTX		105 cases Yang et al. (2017)	U	0.025	U
SLCO1B1*1a (388A/521T)	Methimazole		44 cases, 118 P (Chinese) Jin et al. (2019)	2.21	0.023	1.11–4.39
SLCO1B1*1b (388G/521T)	Methimazole		44 cases, 118 P (Chinese) Jin et al. (2019)	0.52	0.028	0.29–0.93
SLCO1B1*15	Rifampicin		118 cases, 155 P (Chinese) Li et al. (2012)	2.04	0.03	1.05–3.96
SLCO1B1*15	Bosentan		9 cases, 14 P Roustit et al. (2014)	U	0.66	U

OR, odds ratio; P, *p* value; 95%CI, 95% confidence interval; C, medication with adverse reactions; T, medication with no adverse reactions; PC, population control; U, unknown.

#### ABC Transporter

ABC transporter is an ATPase transporter on bacterial plasma membrane that has a very complicated mechanism. In the liver, the elimination of drugs and the excretion of endogenous substances (such as bile acids) both require the participation of ABC transporters (Davidson et al., 2008). Therefore, the genetic polymorphism of the ABC transporter is also a risk factor for DILI, and *ABCC2* and *ABCB1* are of particularly interest.

*ABCB1* is a glycosylated 170-kDa transmembrane protein encoded by the multidrug resistance 1 *(MDR1)* gene and is the most deeply studied drug transporter at present (Zhou et al., 2019). *ABCC2* was first discovered in cisplatin-resistant tumor cells. The multidrug resistance associated protein 2 (MRP2) encoded by *ABCC2* is mainly distributed on the apical membrane of hepatocytes with polarized cells, which is an ATP-dependent amphiphilic anion export pump involved in drug transport and excretion (Baiceanu et al., 2015).

Ethnic differences may exist in the effect of ABC transporters on DILI. In African populations, the single nucleotide polymorphism (SNP) of *ABCB1 rs1045642* can greatly reduce the susceptibility to hepatotoxicity of nevirapine (Ritchie et al., 2006), however, *ABCB1 rs2032582* has taken the place of *ABCB1 rs1045642* in Japanese patients (Fukunaga et al., 2016). *ABCC2 rs717620* mutation can cause the active metabolite of diclofenac to accumulate diclofenac acyl glucuronide in the liver, leading to hepatotoxicity (Daly et al., 2007), but the Spanish population (Ulzurrun et al., 2014) does not support the role of *ABCB1*, *ABCC2* and *ABCB4* transporter gene polymorphisms in the occurrence and development of DILI.

In addition, hepatotoxicity should also be considered in the treatment of cancer patients, especially those who are treated with a combination of analgesics and anticancer drugs. The combination of fentanyl and paclitaxel can inhibit the transport activity of *ABCB1* and cause hepatotoxicity, but the single application of fentanyl does not affect the expression of *ABCB1* (Xie et al., 2015).

#### SLC Transporter

Solute carriers are the second largest membrane protein family in the human genome and contains 52 different gene families. They regulate the uptake or outflow of various basic molecules (such as sugars, amino acids, inorganic ions, neurotransmitters, hormones, vitamins, and drugs) on the cell membrane (Rives et al., 2017). Human genetic data indicate that 50% of SLC family members are associated with human diseases. For DILI, solute carrier organic anion transporter family member 1B1 (*SLCO1B1*) is particularly important.

*SLCO1B1* has an important influence on the absorption, distribution and elimination of endogenous and exogenous substances, especially drugs (Zhang et al., 2019). For anti-TB drugs, in addition to being closely related to drug metabolism enzymes as mentioned above, the mutation of SLCO1B1*15 may also increase the susceptibility to liver injury induced by rifampicin (Li et al., 2012). For cancer patients, except the association between paclitaxel and *ABCB1*, *SLCO1B1 rs4149056* gene variants can affect the toxicity of Chinese non-Hodgkin’s lymphoma patients receiving high dose methotrexate treatment, the hepatotoxicity of TC and CC genotypes was higher than that of TT genotypes (Yang et al., 2017).

### Other

In addition to HLA genes, drug-metabolizing enzymes, ABC transporters and SLC transporters, some polymorphisms in other gene loci are also related to DILI (Table 4).

**TABLE 4:** Other polymorphisms of DILI-related genes identified in GWAS and CGS.

Genes	Drug	Method	Cohort	OR	P	95%Cl
PTPN22 rs2476601	Amoxicillin-clavulanate	GWAS	1,806 cases, 10,397 P (European) Cirulli et al. (2019)	1.62	4 × 10^−6^	1.32–1.98
			133 cases, 1,314 P (African-American)	1.37	1.5 × 10^−6^	1.21–1.56
			109 cases, 718 P (Hispanic Americans) Cirulli et al. (2019)			
NRF2 rs4243387	Anti-TB drugs	CGS	314 cases, 628 P (Chinese) Chen et al. (2019)	1.362	0.038	1.017–1.824
MAFF rs2267373	Anti-TB drugs	CGS	314 cases, 628 P (Chinese) Chen et al. (2019)	0.753	0.025	0.587–0.965
NOS2A rs11080344	Isoniazid	CGS	18 cases, 82 T (Japanese) Nanashima et al. (2012)	0.424	0.043	0.182–0.988
	Rifampicin					
BACH1 rs2070401	Isoniazid	CGS	18 cases, 82 T (Japanese) Nanashima et al. (2012)	016.2	0.018	1.577–166.387
	Rifampicin					
MAFK rs4720833	Isoniazid	CGS	18 cases, 82 T (Japanese) Nanashima et al. (2012)	3.162	0.037	1.033–9.686
	Rifampicin					
XPO1 rs4430924	Anti-TB drugs	CGS	314 cases, 628 T (Chinese) He et al. (2019)	1.938	0.039	1.035–3.628
ERN1 rs199650082	Efavirenz	GWAS	21 cases, 234 T (African) Petros et al. (2017)	18.2	1.4 × 10^−6^	7.1–46.9
lincRNA rs4842407	Efavirenz	GWAS	42 cases, 292 T (African) Petros et al. (2017)	5.4	5.3 × 10^−7^	2.8–10.3

OR, odds ratio; P, *p* value; 95%CI, 95% confidence interval; C, medication with adverse reactions; T, medication with no adverse reactions; PC, population control; U, unknown; Anti-TB drugs, Antituberculosis drugs.

Non-receptor protein tyrosine phosphatase 22 (*PTPN22*) can encode lymphatic tyrosine phosphatase and affect T cell signaling. *PTPN22* polymorphism is associated with a number of autoimmune diseases, including Type I diabetes, Hashimoto’s thyroiditis and so on (Anis et al., 2011). Cirulli et al. (2019) found that except for a few African Americans and Hispanics, non-HLA mutations of *PTPN22 rs2476601* increased the risk of DILI.

In addition, nuclear factor E2-related factor 2 (*Nrf2*) *rs4243387* and *MAFF rs2267373* (Chen et al., 2019), inducible nitric oxide synthase (*NOS2A*), BTB and CNC homology 1 (*BACH1*) and *MAFK* (Nanashima et al., 2012) have been shown to be significantly associated with anti-tuberculosis drug-induced hepatotoxicity in Chinese and Japanese populations. Chinese patients with the nuclear export protein 1 (*XPO1*) *rs4430924* AA genotype were also at significantly higher risk of developing antituberculosis drug-induced hepatotoxicity than those with the GG genotype (He et al., 2019). In antiretroviral therapy, *rs199650082* of nucleus signaling-1 (*ERN1*) is significantly related to DILI, while in antiretroviral therapy combined with anti-tuberculosis therapy, transcriptional variation of synaptotagmin 1 (*Syt1*) *rs4842407* is associated with DILI (Petros et al., 2017). *Syt1* is a long intergene non-coding RNAs (lincRNAs) on chromosome 12, which plays a vital role in endocytosis, exocytosis, and the perception of Ca2+ concentration during vesicle transport; a recent experiment has shown that inhibiting its expression can inhibit the invasion and metastasis of liver cancer (Xu et al., 2020).

## DILI and Epigenomics

The epigenome of a cell is highly dynamic and is controlled by a complex interaction of genetic and environmental factors. Of the many epigenetic markers, DNA methylation (DNAm) is the most stable, and advances in measuring DNAm across the genome have allowed for epigenome-wide association studies (EWAS) (Rakyan et al., 2011). On this basis, existing studies have found a correlation between DNA methylation and the pathogenesis of DILI (Meier and Recillas-Targa, 2017). In addition to the genetic variation of *CYP2D6* and *CYP2E1* mentioned above that may increase the risk of anti-tuberculosis drug-induced liver injury (ATLI), hypermethylation of *CYP2D6* and *CYP2E1* may also increase the risk of liver injury induced by anti-TB drugs (Zhang et al., 2016; Wei et al., 2020). Moreover, methylation on *SLC8A2*, *PSTPIP2* and *AK2* is associated with ATLI (Huai et al., 2019).

## Discussion

DILI-related PGx research not only further indicates the pathogenesis of DILI and its development, but also provides some degrees of reference for the clinical application of DILI, including diagnosis, treatment and prevention.

Differential diagnosis is an important part of the DILI diagnostic scale. The diseases that need to be differentiated include various viral liver diseases, alcoholic liver diseases and other causes. Distinguishing between autoimmune liver disease and DILI is particularly difficult because certain drugs can induce or aggravate autoimmune liver disease. Patients with autoimmune liver disease are also more likely to develop DILI. Autoimmune liver disease and DILI can also be simultaneously existing. The two are mainly identified based on the following 3 points: Firstly, Detailed medical history collection, including the time and frequency of abnormal liver biochemical indicators, whether the two abnormal episodes have completely returned to normal, and the causal relationship between the use of suspected liver injury drugs. Secondly, The histopathological characteristics of liver biopsy. Thirdly, the characteristics of the response to glucocorticoids (Yu et al., 2018). There were many reported cases of liver injury after drug administration, but the causal relationship between drugs and liver injury was not clear. Einar S Bjornsson and Jay H Hoofnagle classified the hepatotoxicity literature of drugs published on the LiverTox website based on the RUCAM scale, which found that about 47% of the drugs had no clear causal relationship with liver injury in the literature (Björnsson and Hoofnagle, 2016). Therefore, it is very likely that many cases of DILI do not meet the definition; hence, a unified analysis based on RUCAM is needed.

Although the RUCAM have a relatively high diagnostic accuracy on DILI, the current diagnostic criteria still lack precision because of the variable onset time, the convert medication association of DILI, and its retrospectiveness rather than prospectiveness. Therefore, the establishment of a detailed DILI diagnostic table is the key to improving the diagnosis rate. At present, some DILI-related susceptibility genes have been incorporated into DILI genomics markers according to Guidelines for the management of DILI (Yu et al., 2017), providing new methods and ideas for DILI diagnosis. In addition, DILI and AIH have similar clinical symptoms in some cases (Chinese Society of Hepatology, Chinese Medical Association, Chinese Society of Gastroenterology, Chinese Medical Association, Chinese Society of Infectious Diseases, Chinese Medical Association, 2016), and it is now possible to enhance the differentiation by sequencing specific HLA alleles (DRB1*03:01 and DRB1*04:01) (de Boer et al., 2017; Zhan et al., 2019). It can be inferred that DILI-related PGx research not only helps to find ideal new biomarkers, but also helps to improve the accuracy of subclinical DILI differential diagnosis. However, the actual application of relevant genetic testing in clinical practice still faces limitations because the low incidence of DILI inevitably leads to a low positive predictive value (PPV) for identified genetic variations, which can also become a future research direction.

Due to the lack of specific treatments for DILI, early identification and rapid discontinuation of suspicious drugs to prevent the development of DILI are currently the most essential and effective solution (Yuan et al., 2017). Therefore, detecting the genotype of patients will help achieve the purpose of precise treatment and provide a theoretical basis for subsequent optimization of treatment plans. The main measures for the prevention of DILI taken at this stage are to avoid or reduce the dosage, but the specific dosage is mostly determined via empirical treatment, and how to accurately administer the dosage still needs to be explored. At present, there are many formulae that can be used to predict the stable dose of warfarin in the population (Xie et al., 2019). Similar to warfarin’s medication formula, combining DILI-related susceptibility gene polymorphisms with medication adverse reaction correlation and clinical data could potentially generate formulae that can effectively avoid the DILI-inducing dosage of various drugs, thereby formulating the benign dosage of different medications. If the susceptible population can be identified before the occurrence of DILI and the individualized hepatotoxicity of drugs can be accurately assessed, it will undoubtedly get twice the result with half the effort. However, current costs of genetic testing are relatively high. From the financial perspective, the scope and application value of genetic screening are still to be discussed.

DILI-related PGx research has demonstrated its unique advantages in clinical applications, but there are still some other fields for DILI related pharmacogenomics study.

Single-gene analysis can only reveal the association between the SNP of a single gene and the disease, leading to shortcomings such as high inherent noise in gene expression profile data and ignored interaction between genes, and hence fails to explain the true relationship between disease-causing genes and diseases. It is a pity that most of the analysis of DILI-related susceptibility genes so far still remains in single-gene analysis, and haplotype analysis for multiple loci is still rare. As a complex disease, DILI requires a comprehensive analysis of various possible susceptibility genes and their associations and even the effects of the correlations between various susceptibility genes on the pathogenesis of DILI. In the future, gene enrichment analysis may be taken into consideration. Genes with the same or similar functions can be defined as gene sets by classifying genes that affect drug metabolism and clearance, and differences among gene sets can be analyzed to draw conclusions closer to the truth. In addition, the possible overlaps of gene sets are also worth noting and may become a breakthrough in future research.

Studies so far have mainly used CGS and GWAS methods. Although these two methods are simple and fast, they still have shortcomings. GWAS has certain requirements for sample size. If the sample size is insufficient, it needs to make up for it through joint meta-analyses. At the same time, GWAS focuses on common gene mutations and the roles of low-frequency genes mutations are prone to be ignored, while low-frequency genes often have a high phenotype and their contribution to disease is basically the same as that of common genes. Considering this problem, it may be possible to introduce high-throughput sequencing based on GWAS research in the future to screen out low-frequency genes related to DILI. For example, in a study of lung cancer susceptibility genes in Chinese Han population (Zhu, 2017), it was found that two low-frequency missense loci in regions *2p23.2* and *7p11.2* were strongly correlated with the prognosis of lung cancer. If the role of new low-frequency genes can be discovered through the combined effect of high-throughput sequencing and GWAS, it may also indicate a new group of key genes and pathways that can affect the prognosis of patients. In addition, animal models can also be established through gene polymorphism to study the activation mechanism of DILI, so as to make the unpredictable DILI predictable. Single-cell RNA sequencing, single-nucleus RNA sequencing and RNA velocity may have advantages in the discovery of new DILI markers, but they still have limitations due to the difficulty of obtaining specimens and low utility in disease prediction. Similarly, the study of epigenetics is therefore limited (Yu et al., 2018). Thus, for patients who are only suspected of DILI and therefore unable to practice a needle biopsy, the DILI serum biomarkers discovered through pharmacogenomics can really be assisted in diagnosis and treatment, such as high mobility group box protein 1, and microRNA-122 (Fontana, 2014). In the future, human induced pluripotent stem cells may be used to establish a liver model of DILI patients without primary cells (Krueger et al., 2014), making research more accessible. In addition, the intervention of gene editing technology can also create more possibilities, such as using CRISPR/dCas9-mediated modification of DNAm to detect the effects of drugs on demethylated and hypermethylated liver cell lines, and to study the association between site-specific DNA methylation and DILI.

At present, the international PharmGKB database has collected 199 drugs, all with identified target genes or metabolic enzyme genes related to efficacy or safety, including 20 molecules and 18 genes related to DILI. However, there is only one article in China containing large-scale DILI epidemiological samples (25,927 cases) (Shen et al., 2019) and PGx research methods had not been used. Meanwhile, the sample size of other similar studies is only two to three hundred people. Perhaps in the future, a DILI case database can be established in China to collect DILI information from each hospital in a unified manner so that researchers can obtain more samples, realize data sharing, and better conduct PGx research. There are more than 1,000 kinds of drugs that can cause DILI in domestic and foreign reports, and related research is still going on now. More relevant databases may appear in the future to meet the urgent needs of scientific researchers and clinicians.

## Conclusion

This article introduces nearly 50 HLA genes, drug metabolizing enzymes, and transporter gene polymorphisms through literature reading, and includes a wide range of drug-induced DILI. Due to the complexity of the mechanisms of DILI, here we introduce a relatively accepted explanation, while the pathogenesis is still inconclusive and needs further research. Understanding the correlation between DILI and related genes is helpful for clinical assessment of the risk of DILI and the auxiliary diagnosis of idiosyncratic DILI, so as to predict and avoid its occurrence. There are still many deficiencies in DILI-related PGx research, however, such as lack of research samples, insufficient studies on Chinese children, and defects in detection and research methods. In the future, it is necessary to conduct in-depth DILI-related PGx studies and expand relevant databases domestically and abroad in order to obtain more conclusions. And it is hoped that through these conclusions, better methods can be studied and applied to the clinic in order to more effectively reduce the risk of idiosyncratic DILI.
